# Elagolix as a Diagnostic and Therapeutic Bridge to Surgery in Refractory Progestogen Hypersensitivity

**DOI:** 10.1210/jcemcr/luaf314

**Published:** 2025-12-30

**Authors:** Ali Osman Balkan, Divya Sistla, Erin Lyn Rhinehart

**Affiliations:** Department of Internal Medicine, University of Pittsburgh Medical Center, Pittsburgh, PA 15213, USA; Division of Endocrinology, University of Pittsburgh Medical Center, Pittsburgh, PA 15213, USA; Department of Obstetrics and Gynecology, University of Pittsburgh Medical Center, Pittsburgh, PA 15213, USA

**Keywords:** progesterone, progestogens, oophorectomy, hormonal allergy

## Abstract

Progestogen hypersensitivity (PH) is a rare and underrecognized condition characterized by cyclical allergic reactions to endogenous or exogenous progestogens. We present a 27-year-old woman with an 11-year history of recurrent luteal-phase urticaria, angioedema, and anaphylaxis refractory to antihistamines, corticosteroids, and hormonal suppression. She was diagnosed with PH at age 21. Introduction of elagolix, a GnRH antagonist, led to marked symptomatic improvement by suppressing ovarian hormone production. Due to cost and side effects, definitive surgical management with total laparoscopic hysterectomy and bilateral salpingo-oophorectomy was performed, resulting in complete remission. Fewer than 200 cases are reported in the literature, and standardized diagnostic or treatment protocols are lacking. This case highlights the diagnostic complexity of PH, the therapeutic potential of GnRH antagonists as a bridge to surgery, and the importance of a multidisciplinary approach. It underscores the need for greater awareness, standardized diagnostic criteria, and further research into the immunologic mechanisms underlying PH to guide future management strategies.

## Introduction

Progesterone hypersensitivity (PH) is a hypersensitivity reaction to either endogenous progesterone or exogenous progestins. It presents as recurrent, cyclical cutaneous or systemic symptoms during the luteal phase, coinciding with peak progesterone levels. It is exceptionally rare, with fewer than 200 cases reported in the literature [1]. Recent consensus statements from the European Academy of Allergy and Clinical Immunology and the American Academy of Allergy, Asthma, and Immunology describe PH as a heterogeneous disorder with variable manifestations. Both organizations emphasize that diagnosis is primarily clinical, based on the temporal relationship between symptoms and progestogen exposure [1, 2]. Immunopathogenesis of this condition exhibits heterogeneity and remains incompletely characterized; however, both type I (IgE-mediated) immediate hypersensitivity and type IV (T-cell-mediated) delayed hypersensitivity mechanisms have been implicated [2]. Clinical features include urticaria, angioedema, eczema, erythema multiforme, vesiculobullous eruptions, and, in severe cases, anaphylaxis occurring 3 to 10 days before menses and resolving after onset [3]. The rarity, underrecognition, and variability of presentations often delay diagnosis and management. Early recognition is critical to prevent recurrent symptoms and improve quality of life. This case describes a young woman with severe, refractory PH who achieved remission after total laparoscopic hysterectomy and bilateral salpingo-oophorectomy (TLH-BSO). Early onset, refractoriness, elagolix use, and young age at surgery distinguish it from most reported cases, which typically involve older women and rarely document elagolix or early definitive surgery [1, 4, 5]

## Case Presentation

**Figure 1.**
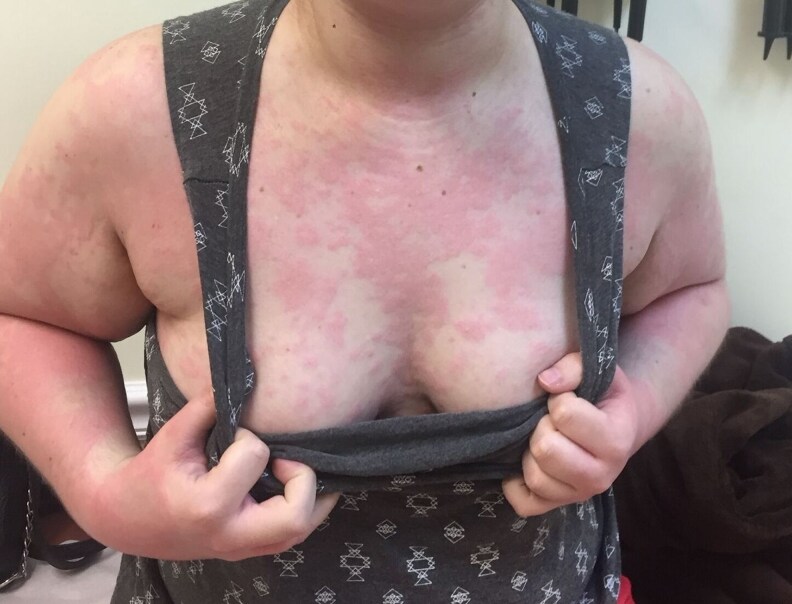
Erythematous, patchy, and partly confluent lesions over the chest, upper arms, shoulders, and inframammary region. Broad and symmetric involvement is characteristic of progestogen hypersensitivity, especially in the luteal phase.

**Figure 2.**
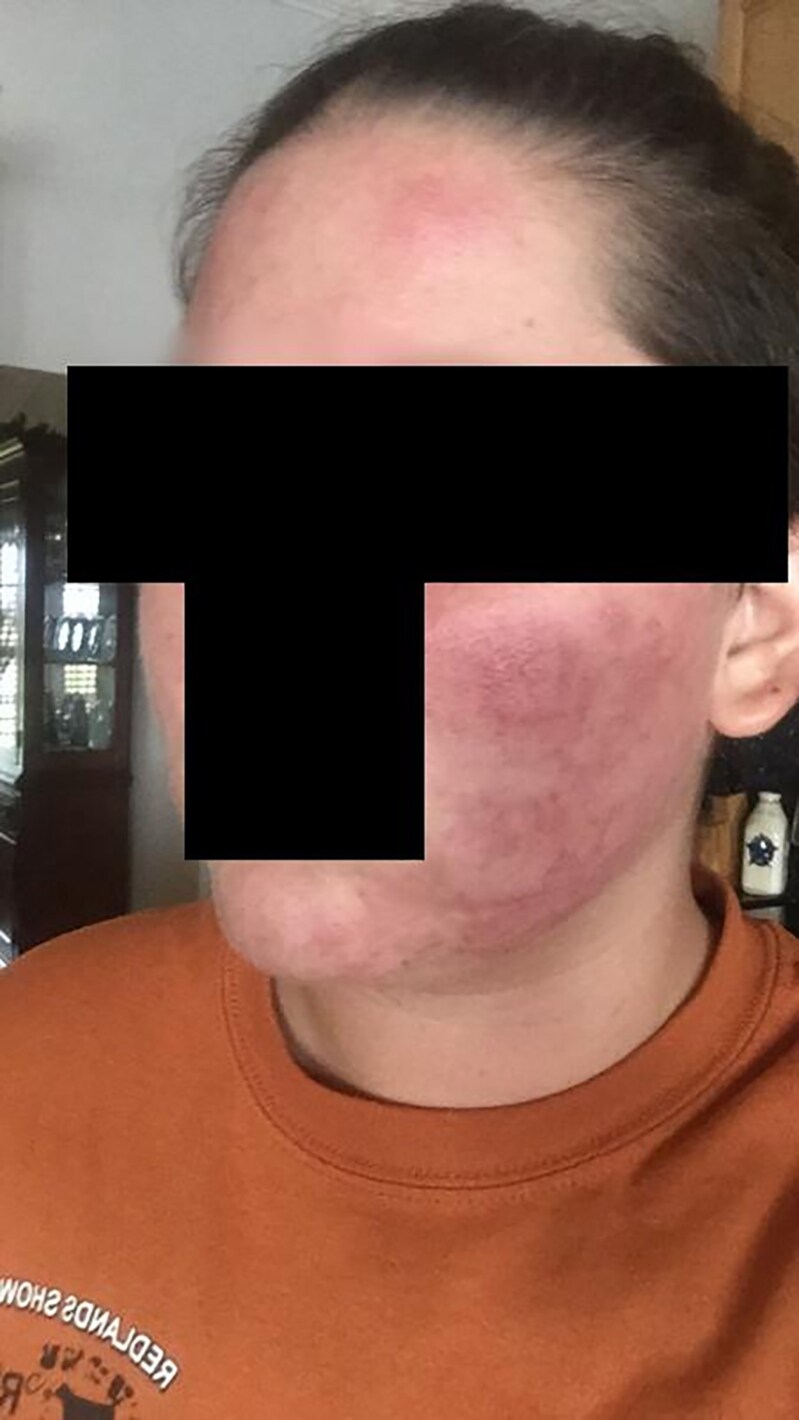
Erythematous and inflamed lesions over the forehead and bilateral malar areas. This facial distribution may mimic rosacea or lupus but, in the context of cyclic flares, supports a diagnosis of progestogen hypersensitivity.

**Figure 3.**
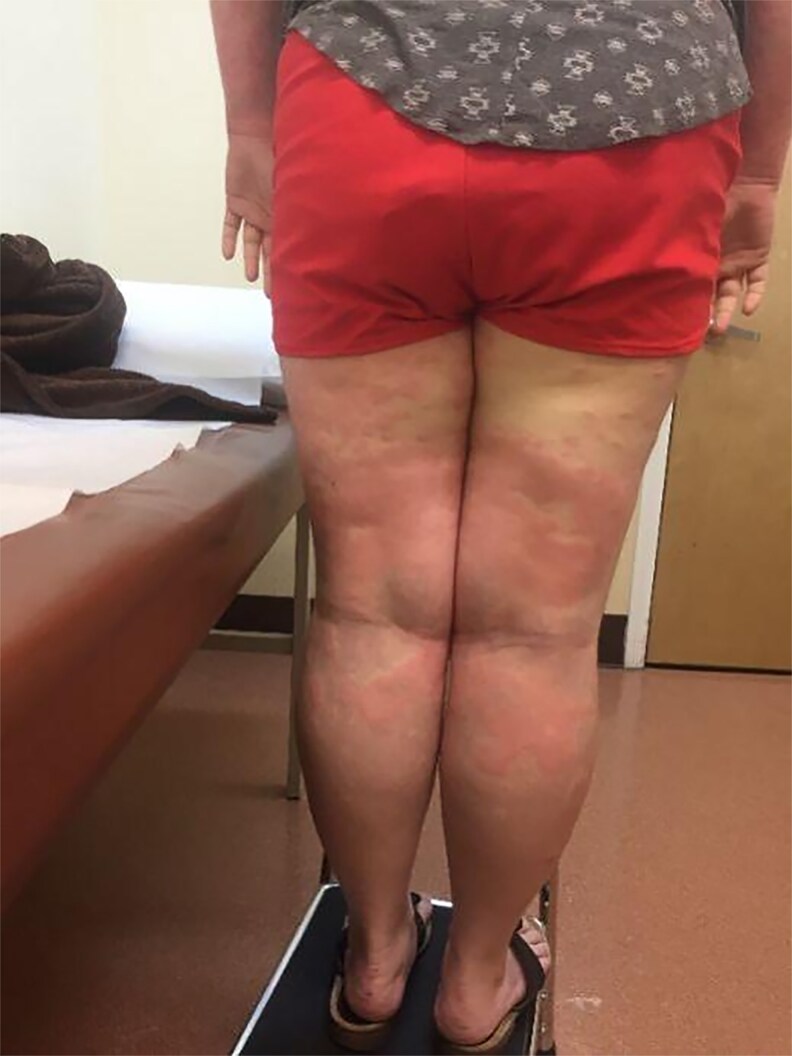
Inflamed patches that resemble urticarial or eczematous eruptions are commonly seen in progestogen hypersensitivity.

**Figure 4.**
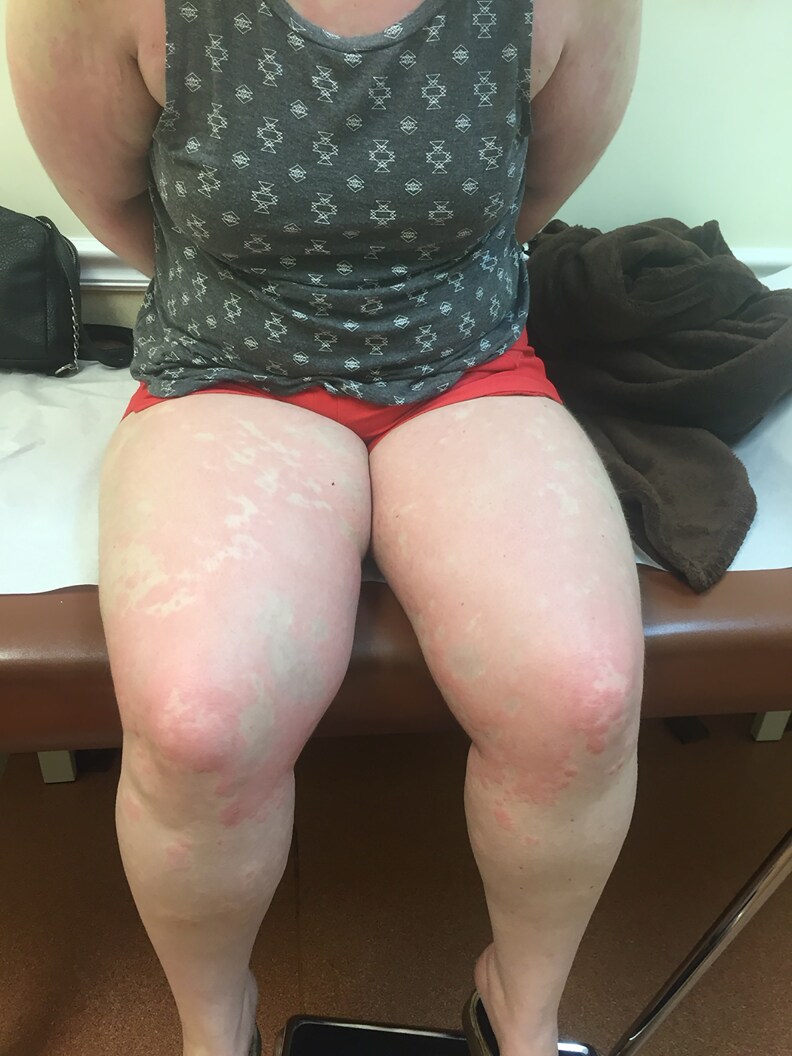
Bilateral, symmetric erythematous, and hypopigmented plaques on the thighs and knees, with irregular borders and some confluence.

**Figure 5.**
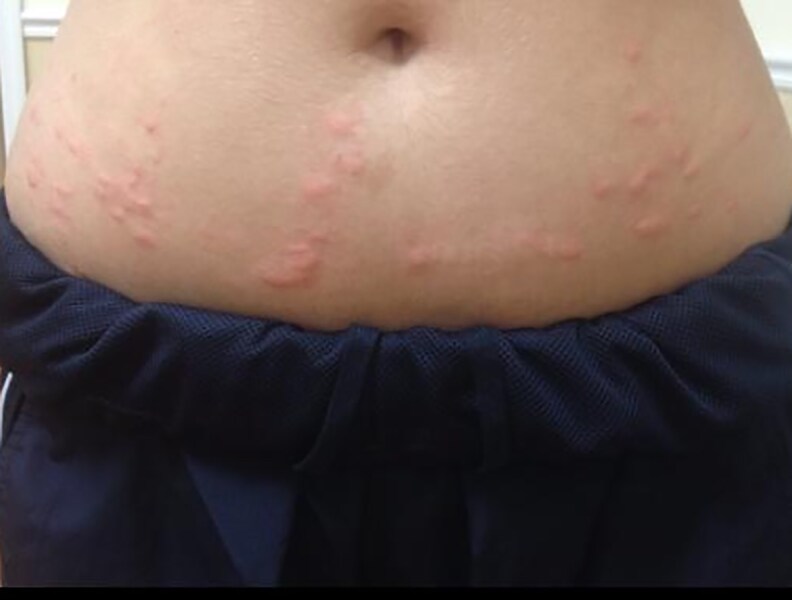
Symmetrical distribution of erythematous, raised papules, and plaques localized to the lower abdominal region. Bilateral symmetry suggests systemic etiology rather than localized trauma or infection.

**Figure 6.**
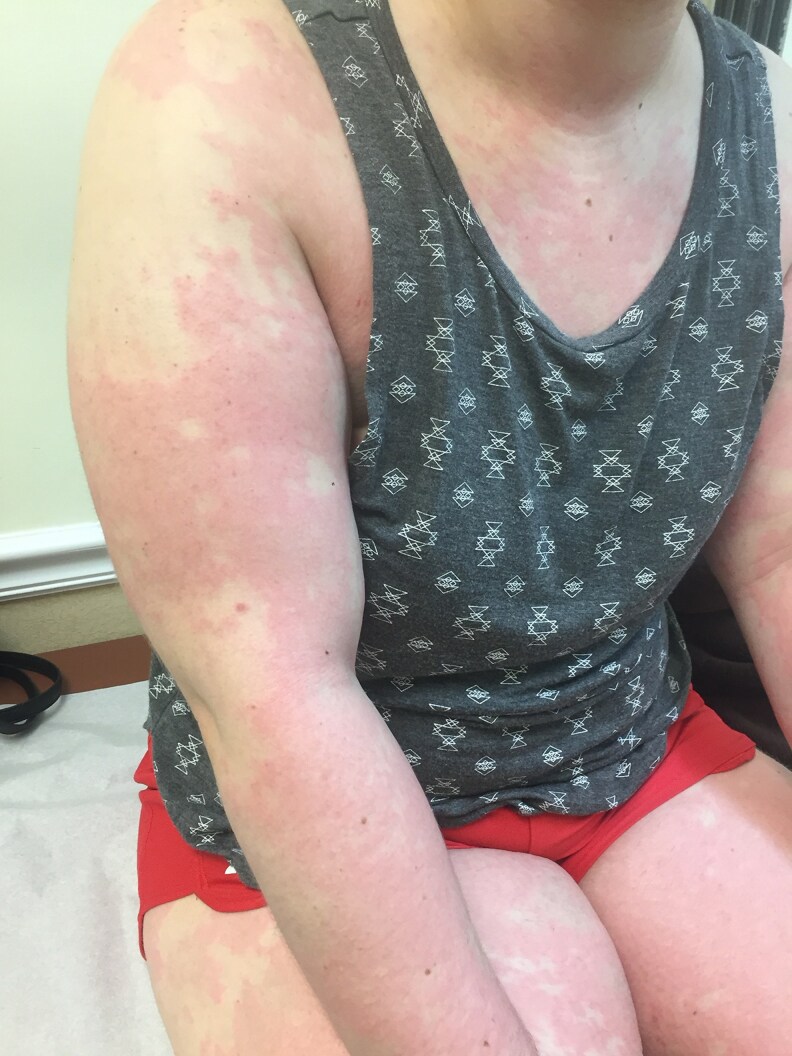
Extensive, confluent erythematous and papular eruption with scattered areas of relative sparing and hypopigmentation, primarily involving the upper extremities and torso.

A 27-year-old Caucasian nulliparous woman with hypothyroidism presented with an 11-year history of cyclic urticaria and anaphylaxis (Table 1). Symptoms began at age 16, shortly after initiating oral contraceptive therapy with drospirenone/ethinyl estradiol for acne; rather than improving her condition, the medication coincided with the onset of hives, and her episodes included generalized urticaria, facial and body angioedema, and multiple anaphylactic events, consistently occurring during the luteal phase and resolving after menstruation (Figs. 1-6). On 1 occasion, hives persisted for a week and progressed to facial swelling, dyspnea, and chest tightness, requiring 2 intramuscular epinephrine doses at urgent care. She eliminated common allergens and kept detailed records to identify triggers. After years of severe symptoms, she noted a clear temporal correlation between episodes and her menstrual cycle.

**Table 1.** Timeline of symptoms, diagnosis, and management

Age	Event/symptom	Intervention/treatment	Outcome
16-20 years	Onset of recurrent luteal phase urticaria, angioedema, and anaphylaxis	Symptomatic treatment with antihistamines and corticosteroids	Partial relief; symptoms persisted
21 years	Diagnosis of progestogen hypersensitivity	Confirmed clinically; continued symptomatic management	Symptoms continued
23-26 years	Severe refractory symptoms	Introduction of elagolix (GnRH antagonist)	Marked improvement but side effects and cost issues
27 years	Decision for definitive management	TLH-BSO	Complete remission

Abbreviations: TLH-BSO, total laparoscopic hysterectomy and bilateral salpingo-oophorectomy.

## Diagnostic Assessment

Skin prick testing was attempted but was inconclusive due to baseline urticaria. Punch biopsy of a neck lesion revealed bland spindle cells in a whorled pattern; immunohistochemistry showed diffuse CD34 positivity and negativity for factor XIIIa, pan-cytokeratin, and S100, consistent with benign myofibroblastic proliferation (eg, nodular fasciitis). The patient was prescribed a combined oral contraceptive pill to manage symptoms and subsequently developed generalized urticaria on multiple occasions. A clinically observed rechallenge in a gynecology office, initiated at the patient's request, reproduced symptoms within 45 minutes, confirming a positive oral progestogen challenge. She was diagnosed with hypothyroidism at age 18 and tested positive for thyroid peroxidase antibodies at a level of 664 U/mL (SI: 664 IU/L) (reference range <61 U/mL [SI: <61 IU/L]) but negative for thyroglobulin antibodies. Thyroid function tests remained within normal limits: TSH 1 to 2 mIU/L (SI: 1-2 mIU/L) (reference range 0.4-4.0 mIU/L [SI: 0.4-4.0 mIU/L]) and free T4 1.5 ng/dL (SI: 19.3 pmol/L) (reference range, 0.8-1.8 ng/dL [SI: 10-23 pmol/L]). At age 27, antinuclear antibodies were positive at 1:320 during evaluation for joint pain. The rheumatoid factor was <10 IU/mL (reference range, <14 IU/mL); the erythrocyte sedimentation rate was 18 mm/hr (reference range, <20 mm/hr); and the C-reactive protein concentration was <1.0 mg/dL (SI: <10 mg/L) (reference range <1.0 mg/dL [SI: <10 mg/L]). Diagnosis was based on a consistent temporal relationship between symptoms and the luteal phase, reproducible hypersensitivity to exogenous progestin, and symptom resolution with ovarian suppression (elagolix).

## Treatment

Initial management focused on symptomatic relief and allergen avoidance, which were largely ineffective. Initial management with antihistamines (cetirizine, levocetirizine, and ranitidine), doxepin, and montelukast provided only partial relief. Dietary changes, including paleo, gluten-free, and plant-based diets, yielded partial benefits only on a strict plant-based regimen. Given the clear luteal-phase symptom correlation, hormonal suppression was pursued. Combined oral contraceptives triggered immediate hypersensitivity. Elagolix, an oral GnRH antagonist, was initiated at 150 mg daily, inducing amenorrhea and a 9-month symptom-free interval. Adverse effects included vasomotor symptoms, sleep disturbances, and vaginal dryness. After breakthrough ovulation with generalized urticaria, the dose was increased to 200 mg twice daily, restoring amenorrhea and substantial symptom control. However, the high cost, lack of Food and Drug Administration approval for this indication, and concerns regarding long-term hypoestrogenic effects such as osteoporosis made elagolix unsustainable. She declined desensitization and long-term hormonal therapy due to a history of anaphylaxis and concerns regarding safety and fertility. She considered pregnancy-related progesterone surges life-threatening and expressed a desire for permanent sterility and a childfree lifestyle. Fertility preservation (oocyte stimulation, cryopreservation, embryo freezing) was refused because of hormonal exposure risks. After multidisciplinary counseling and informed consent emphasizing the irreversible nature of TLH-BSO, she underwent the procedure at age 27.

## Outcome and Follow-up

Postoperatively, elagolix was discontinued and transdermal estrogen was initiated to mitigate surgical menopause risks. She remains entirely asymptomatic with no recurrence of urticaria, angioedema, or anaphylaxis, reporting a marked improvement in her quality of life. Routine annual gynecologic follow-up for hormone replacement management is planned until approximately age 50. Bone mineral density surveillance has been initiated, and her most recent dual-energy X-ray absorptiometry scan was normal.

## Discussion

Autoimmune progesterone dermatitis historically described cyclical luteal-phase eruptions. Foer et al [4] proposed “progestogen hypersensitivity” to encompass reactions to endogenous progesterone and synthetic progestins, reflecting heterogeneity and mechanistic uncertainty. PH is rare and often misdiagnosed, triggered by endogenous fluctuations or exogenous progestins [4, 6]. In 58% of cases, symptoms begin after exogenous exposure [4]. Our patient had no atopy history and developed hives after oral contraceptives, with symptoms 3 to 4 days before menses that resolved after onset.

Diagnosis is challenging because of heterogeneity and the absence of standardized criteria. Progesterone-specific IgE assays and skin testing offer limited accuracy, making clinical assessment based on symptom timing and hormone exposure the primary approach. The American Academy of Allergy, Asthma, and Immunology emphasizes correlating symptom chronology with the menstrual cycle and supports intracutaneous progesterone testing, though its specificity remains debated [6, 7]. This test involves a 0.1 mL intradermal progesterone injection; however, false positives are common in healthy controls due to nonspecific irritant effects [7]. Our patient was evaluated by an allergist. Skin prick testing was inconclusive as she had hives at the time of test. Progesterone challenge is another consideration in select cases to confirm the diagnosis when history is unclear or exogenous PH is suspected [1]. The challenge should occur in a controlled setting with precautions due to risk of severe reactions, including anaphylaxis [2]. Her positive oral progesterone challenge supported the diagnosis, along with symptom chronology, response to elagolix, and remission post-oophorectomy. Her thyroid peroxidase and antinuclear antibodies positivity were incidental; PH is a distinct immunologic disorder mediated by IgE or T-cell responses and is unrelated to thyroid autoimmunity [4, 5, 8, 9].

Differentials include catamenial anaphylaxis (symptoms at menstruation), chronic urticaria (persistent, less hormone-linked), and autoimmune estrogen dermatitis (follicular-phase flares) [10]. Chronic urticaria with luteal flares may worsen periodically but lacks consistent progesterone association and the clear cyclicity of PH. Unlike chronic urticaria, which is generally persistent, PH shows predictable eruptions such as urticaria, angioedema, or anaphylaxis occurring 3 to 10 days before menses, resolving after onset [3]. Autoimmune estrogen dermatitis is unlikely, as symptoms typically worsen during the follicular phase and do not explain reactions to exogenous progesterone.

Optimal PH management requires a multidisciplinary approach to address physiological and psychosocial needs. For mild to moderate PH, initial therapy targets symptoms. Second-generation H1-antihistamines may control mild cyclical urticaria or pruritus [10]. Ovulation suppression to reduce endogenous progesterone is a mainstay of management, but combined oral contraceptives may not be suitable for all patients due to potential hypersensitivity to exogenous progestins [2, 11]. Alternatives include GnRH agonists (eg, leuprolide), which can induce hypoestrogenism and suppress ovulation [2]. For patients requiring high-dose progestins for fertility treatment and in vitro fertilization, Foer et al [4] recommend rapid intramuscular progesterone desensitization; in their study, all 3 patients tolerated in vitro fertilization. In severe, refractory cases of endogenously triggered PH, bilateral oophorectomy is considered curative [1] and is typically reserved for individuals who have completed childbearing. Our patient underwent TLH-BSO at 27, with complete symptom resolution and no adverse effects. A BSO alone would not have sufficed for symptom management, as uterine retention would have necessitated exogenous progestins for endometrial protection during hormone replacement therapy. This consideration was central to selecting the surgical approach that aligned with the patient's goals. Surgical menopause after early oophorectomy necessitates long-term follow-up to manage associated risks of osteoporosis and cardiovascular disease, especially before age 45 [12].

Pregnancy in PH shows variability, with cases of resolution, persistence, or exacerbation. Huang et al [13] describe a patient whose symptoms disappeared during pregnancy and recurred after miscarriage. In Foer et al's 24-case series [4], pregnancy outcomes varied: some women required desensitization protocols to tolerate fertility treatments and maintain pregnancy, with most achieving symptom control. In other case reports [14, 15] of patients with severe hypersensitivity to progesterone, oophorectomy was also ultimately the chosen management strategy and was curative.

A unique aspect of this case, compared to previously reported cases [2, 4, 6, 16-19] (Table 2), is the use of elagolix. Elagolix is an oral GnRH antagonist approved for moderate to severe endometriosis pain [20]. It competitively inhibits pituitary GnRH receptors, causing dose-dependent suppression of LH and FSH, which lowers ovarian sex hormones, including estradiol and progesterone [20]. At 150 mg daily, ovulation suppression occurs in ∼50% of patients; 200 mg twice daily achieves greater suppression [21]. Our patient experienced a prolonged symptom-free period, then 1 flare on 150 mg; increasing to 200 mg twice daily achieved complete remission. Because of significant side effects, high costs (∼$1200/month), and patient preference for definitive treatment, surgery was pursued.

**Table 2.** Summary of some of the reported cases and series of progestogen hypersensitivity

Author/year	Patient demographics	Trigger (endogenous/exogenous)	Clinical manifestations	Diagnostic approach	Treatments tried	Outcomes/note
Li et al, 2018 [2]	Review, mostly women of childbearing age	Both	Urticaria, angioedema, anaphylaxis, non-evanescent eruptions	History, skin test, challenge	Ovulation suppression, desensitization, surgery	Successful management; higher prevalence expected
Foer et al, 2016 [4]	24 women, median age 34, 42% atopic	42% endogenous, 58% exogenous	Cyclical dermatitis, urticaria, angioedema, asthma, anaphylaxis	History, skin test, challenge	Antihistamines, steroids, GnRH agonists, desensitization, surgery	Symptom control in most; IVF tolerance in 3; 2 pregnancies postdesensitization
Bernstein, 2020 [6]	Adult woman	Endogenous and exogenous	Urticaria, angioedema, dermatitis, anaphylaxis	History, progesterone-specific IgE	Antihistamines, ovulation suppression, desensitization	Successful management; highlights diagnostic algorithm
Claffey et al, 2024 [16]	14-year-old, atopic	Endogenous	Erythema multiforme, mucositis	Multidisciplinary workup	JAK inhibitor, hormonal suppression	Remission with JAK inhibitor after hormonal therapy failure
Patel et al, 2023 [17]	Review, various ages	Both	Erythema multiforme, eczema, urticaria, angioedema, asthma, anaphylaxis	History, skin test, ELISA	Ovulation suppression, desensitization	Symptom control; increased recognition
Foer and Buchheit, 2019 [18]	Literature review, various ages	Both	Dermatitis, urticaria, asthma, anaphylaxis	History, test results	Ovulation suppression, desensitization	Variable: remission, recurrence, or partial remission
Chiarella et al, 2023 [19]	Adolescent female	Endogenous	Urticaria, anaphylaxis	History, skin test	GnRH agonist, antihistamines, steroids	Remission with ovulation suppression; diagnostic challenge

Abbreviations: IVF, in vitro fertilization; JAK, Janus kinase.

Key limitations include the following: this single case may not be broadly applicable given PH's rarity and variability. No diagnostic gold standard exists, and diagnosis often relies on clinical history. Progesterone-specific IgE assays remain scarce and have limited accuracy. Treatment options including GnRH antagonists like elagolix, desensitization protocols, and surgery are constrained by cost, insurance, and accessibility. In summary, although elagolix is a mechanistically rational option for PH, broader clinical use requires research to address efficacy, safety, and practical considerations. Further research should clarify immunopathogenic mechanisms, particularly the role of progesterone and progestins in cutaneous inflammation. Well-designed studies are needed to address heterogeneity, refine diagnostic criteria, and guide targeted therapies, including GnRH antagonists (eg, elagolix).

## Learning Points

- PH is a rare, cyclical immune-mediated reaction with diverse manifestations. Diagnosis remains challenging due to the absence of standardized criteria and guidelines; emerging biomarkers such as progesterone-specific IgE, cytokine profiles, and T-cell assays may improve accuracy but require validation.
- Elagolix, a GnRH antagonist, may serve as a diagnostic and therapeutic bridge by suppressing ovulation, though its use is off-label and limited by cost and safety concerns.
- PH imposes a significant psychosocial burden and reduces quality of life due to recurrent allergic reactions and unpredictable symptoms. Multidisciplinary care involving allergy/immunology, gynecology, endocrinology, and psychiatry is essential.

## Contributors

All authors made individual contributions to the manuscript. E.R.: diagnosis and management of the patient and revision of the manuscript. A.O.B.: conceptualization of the report; acquisition, analysis, and interpretation of data; preparation of the original manuscript; and revision of the manuscript. D.S.: diagnosis and management of the patient, conceptualization of the report, drafting of the manuscript, and revision of the manuscript. All authors critically reviewed and approved the final draft.

## Data Availability

Data sharing is not applicable to this article as no data sets were generated or analyzed during the current study.
